# Anti-Arthritic and Anti-Cancer Activities of Polyphenols: A Review of the Most Recent In Vitro Assays

**DOI:** 10.3390/life13020361

**Published:** 2023-01-28

**Authors:** Muhammad Ali, Viviana Benfante, Alessandro Stefano, Anthony Yezzi, Domenico Di Raimondo, Antonino Tuttolomondo, Albert Comelli

**Affiliations:** 1 Ri.MED Foundation, Via Bandiera 11, 90133 Palermo, Italy; 2 Department of Health Promotion, Mother and Child Care, Internal Medicine and Medical Specialties, Molecular and Clinical Medicine, University of Palermo, 90127 Palermo, Italy; 3 Institute of Molecular Bioimaging and Physiology, National Research Council (IBFM-CNR), 90015 Cefalù, Italy; 4 Department of Electrical and Computer Engineering, Georgia Institute of Technology, Atlanta, GA 30332, USA; 5 NBFC—National Biodiversity Future Center, 90133 Palermo, Italy

**Keywords:** polyphenols, rheumatoid arthritis, cancer, in vitro, metastasis, signaling cascade, cellular models, therapeutic adjuvant

## Abstract

Polyphenols have gained widespread attention as they are effective in the prevention and management of various diseases, including cancer diseases (CD) and rheumatoid arthritis (RA). They are natural organic substances present in fruits, vegetables, and spices. Polyphenols interact with various kinds of receptors and membranes. They modulate different signal cascades and interact with the enzymes responsible for CD and RA. These interactions involve cellular machinery, from cell membranes to major nuclear components, and provide information on their beneficial effects on health. These actions provide evidence for their pharmaceutical exploitation in the treatment of CD and RA. In this review, we discuss different pathways, modulated by polyphenols, which are involved in CD and RA. A search of the most recent relevant publications was carried out with the following criteria: publication date, 2012–2022; language, English; study design, in vitro; and the investigation of polyphenols present in extra virgin olive, grapes, and spices in the context of RA and CD, including, when available, the underlying molecular mechanisms. This review is valuable for clarifying the mechanisms of polyphenols targeting the pathways of senescence and leading to the development of CD and RA treatments. Herein, we focus on research reports that emphasize antioxidant properties.

## 1. Introduction

Plants have been used as medicines for more than 5000 years to treat a variety of diseases in humans. These phytomedicines have been able to cure animals and humans due to their beneficial natural phytochemicals. Phytochemicals are secondary metabolites of plant sources and have unique bioactive organic compounds with multiple pharmacological activities, including antiviral, anti-inflammatory, antineoplastic, and antioxidant. These phytochemicals include polyphenols [1], which are naturally occurring organic compounds in grapes, berries, nuts, olives, coffee, tea, flaxseed, and spices such as rosmarinus officinalis, origanum vulgare, salvia officinalis, and majorana syriaca [2]. The chemical structure of polyphenols consists of aromatic rings to which one or more hydroxyl groups are attached. Flavonoids, xanthones, catechin, hesperetin, quercetin, ellagic acid, lignans, stilbenes, chalcones, polyphenolic amides, and resveratrol fall within the class of polyphenolic substances [3,4].

Polyphenols possess anticancer and anti-inflammatory properties which modulate signaling pathways, induce apoptosis in various kinds of cancer cells, and reduce nucleoside diphosphate kinase B activity in lung, bladder, and colon cancer cells. Nucleoside diphosphate kinase B (NME2) plays an important role in many cellular processes. As a transcription factor, NME2 acts on the oncogene c-MYC, which is involved in the development of cancer [5].

Similarly, in recent decades, medicinal plants have been investigated for anticancer activity. These phytochemicals play a crucial role in maintaining the molecular signaling pathways of cancer. They can inhibit fatty acid synthesis, topoisomerase I/II suppression and downregulation, p53 accumulation, cell cycle arrest, proteasome inhibition, and regulation of survival/proliferation events. Unlike common approaches to cancer treatment that require lengthy and painful procedures, they have fewer side effects. The development of allopathic drugs requires complex procedures, from selecting a molecule to the production of medicines that might lead to unexpected failure, side effects, and toxicity [6]. Thus, researchers are continuously searching for novel alternative strategies for dealing with such conditions in a more effective way.

Polyphenols inhibit cell proliferation and cell cycle arrest by suppressing the nuclear factor kappa-B (NF-kB) pathway [7]. Moreover, their biological activities have beneficial effects on the treatment of neurodegenerative disorders, cardiovascular disease (CD), Type 2 diabetes (T2D), obesity, cancer, and rheumatoid arthritis (RA) [8].

RA is an inflammatory, chronic autoimmune disease that starts by affecting small joints, and cartilage, further progressing to larger joints. Skin, eyes, heart, kidneys, and lungs are eventually affected, and in some cases, bones, ligaments, and tendons can be damaged. The major symptoms of RA are swollen and warm joints, fatigue, fever, weight loss, and rheumatoid nodules under the skin [9]. Approximately 1% of the world’s population is diagnosed with RA, which affects more women than men [10]. The commonly prescribed anti-rheumatic drugs are Tumor Necrosis Factor (TNF) inhibitors, non-steroidal anti-inflammatory drugs (NSAIDs), IL-6 inhibitors, steroids, methotrexate, and hydroxychloroquine [8]. Nevertheless, these drugs have various side effects such as elevated cholesterol, neutropenia, lymphopenia, elevation of transaminase, cytopenia, and gastrointestinal side effects [11]. However, these treatments do not provide proper relief for patients, and there is no proper treatment for RA. Research is ongoing to discover new anti-arthritis drugs by designing new phytomedicines with fewer side effects and less toxicity [12].

This review article is a collection of recent and relevant papers from 2012–2022, aimed at research seeking information on this topic. We used the PubMed and Google Scholar databases to search for appropriate, focused, and accessible data. The main objective of the current review paper is to discuss the antitumor and anti-inflammatory properties of polyphenols present in olive oil, spices, and grapes that have been studied in cancer and RA cell lines. Furthermore, the reported literature elucidates the modulatory effects of polyphenols such as resveratrol, hydroxytyrosol, and quercetin on cancer and RA cell lines. We also assessed new therapeutic approaches that could be used in RA and cancer treatment.

## 2. Methodology

This study consists of an up-to-date review of the literature addressing the in vitro anti-tumor and anti-inflammatory effects of plant extracts rich in polyphenols and polyphenolic compounds, originating in food and herbal medicine.

Criteria for selecting the material were as follows: a search was conducted on PubMed and Google Scholar database using the keywords “cancer AND polyphenols AND in-vitro”, and “arthritis AND polyphenols AND in-vitro”.

The time range was selected as 2012 to 2022. The three main mechanisms for cell apoptosis, biological effects, pharmacological effects, and phytochemistry of the various plant extracts in their traditional usage were also considered. Conference abstracts and book chapters were excluded.

## 3. Biological Basis of Rheumatoid Arthritis

RA is a chronic autoimmune inflammatory joint disease that causes cartilage and bone damage. Initially, it affects the joints, but it progresses to the eyes, kidneys, skin, lungs, and heart [7]. The major symptoms of RA are swollen and warm joints, fatigue, fever, weight loss, and rheumatoid nodules under the skin [9]. The factors that initiate the cause of RA are poorly understood. Genetic factors, including class II major histocompatibility antigens/human leukocyte antigens (HLA-DR) and non-HLA genes, play a crucial role in the pathogenesis of RA. The HLA and a few non-HLA genes have also been linked to citrullinated proteins called anti-citrullinated protein antibodies (ACPA) [13]. Anti-citrullinated protein antibodies (ACPAs) are autoantibodies that attack peptides and proteins that contain citrulline. As a result of inflammation, the arginine amino acid in proteins can be converted into citrulline by the calcium-dependent enzyme peptidyl-arginine-deiminase (PAD); this process is called citrullination. When the shape of proteins is altered, the immune system recognizes those proteins as antigens and initiates a response [14]. Exposure to air pollution, including silica dust, smoke, and carbon-derived nanomaterials, can stimulate mucosal toll-like receptors (TLRs) that activate PADs and antigen-presenting cells (APCs). Citrullinated proteins are triggered by smoking in the context of the HLA-DR SE gene [15]. There are also pathogens that trigger RA, such as *Aggregatibacter actinomycetemcomitans* (Aa) and *Porphyromonas gingivalis* [16]. As a result of *P. gingivalis* infection, ACPA is produced in two ways: the first is by the formation of neutrophil extracellular traps (NETs) during NETosis, and the second is by arginine gingipains proteins and PAD that cleave the protein at arginine residues, which, therefore, produces citrullinated proteins that are more immunogenic [17]. The *Aa* infectious agent releases leukotoxin A and creates a pore in the membrane of neutrophils. This causes hyper-citrullination of neutrophils and the release of citrullinated autoantigens [16].

The pathobiology of RA involves the innate and adaptive immune systems. Cytokines are released by the body, causing the expression of adhesion molecules on the synovium membrane to increase. This allows inflammatory cells such as lymphocytes, macrophages, and plasma cells to migrate to the synovium, where they multiply and activate similar fibroblast-like synoviocytes (FLS). FLS stimulates the expression of the receptor activator of nuclear factor kappa-B ligand (RANKL), and, consequently, a high expression of pro-inflammatory cytokines occurs, leading to the initiation of osteoclast activity. Osteoclast cells absorb bone tissues and eventually, bone erosion occurs. FLS cells also release matrix metalloproteinases (MMPS), which are proteases responsible for the degradation of cartilage. Inflammation of the synovial membrane and the presence of inflammatory cells cause synovial hyperplasia and pannus formation, which consists of the extra growth of the joints and cartilage. High expression of vascular endothelial growth factor (VEGF) causes angiogenesis, resulting in more blood flowing to assist the maintenance of the pannus. The formation of the pannus leads to chronic inflammation and is responsible for the production of collagenase and protease [18].

Reactive oxygen species (ROS) also contribute to RA development. This is due to the fact that macrophages activated by excessive production of proinflammatory cytokines secrete reactive oxygen species (ROS), leading to the destruction of cartilage and joints [10,11].

The innate immune system is the first line of defense against pathogens. Many factors play a role in innate immunity, such as antibacterial peptides, mannose-binding lectins, the alternate pathway of complement activation, and cytokines. Dendritic cells, neutrophils, macrophages, T lymphocytes, and natural killer cells have major roles in the host’s immune response. Macrophages play a key role in the progression of RA, releasing inflammatory cytokines such as Interleukin 1 beta (IL-1β), Interleukin 6 (IL-6), tumor necrosis factor alpha (TNF-α), matrix-degrading enzymes, and reactive oxygen species. Furthermore, macrophages also stimulate and proliferate FLS [19].

**Figure 1:**
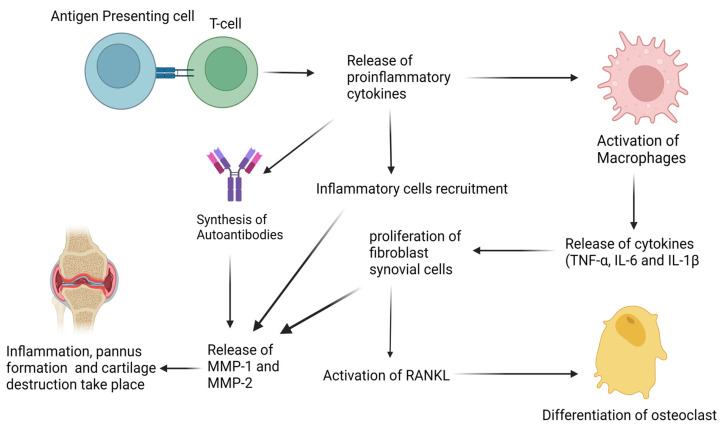
Schematic pathogenesis of RA. The process of immune activation and disease progression involves the activation of both the innate and adaptive immune systems.

TNF-α is an inflammatory cytokine released by immune cells such as macrophages, natural killer cells, endothelial cells, activated lymphocytes, and neutrophils (Figure 1). It mediates the activation, migration, and adhesion of immune cells, and contributes to the processes of angiogenesis and osteoclastogenesis [20].

IL-6 is produced by FLS cells and macrophages. It stimulates endothelial cells to release chemokines and activates B and T cells, as well as osteoclasts. IL-6 plays an active role in the production of VEGF, which is responsible for pannus formation. IL-1β is produced in the inflamed synovium by macrophages and monocytes, and has the same activities as TNF-α [21]. These inflammatory cytokines induce inflammation by activating various pathways such as mitogen-activated protein kinase (MAPks), c-Jun N-terminal kinase (JNK), extracellular signal-regulated kinase (ERK), p38, phosphatidylinositol 3-kinase (PI3K)/protein kinase B (AKT), and NF-κB, responses that are not antigen-specific, adaptive, or associated with immunologic memory. Inflammatory markers such as IL-6, IL-10, the neutrophil-to-lymphocyte ratio (NLR), the platelet-to-lymphocyte ratio (PLR), TNF-α, and C-reactive protein (CRP) play important roles in the pathogenesis of RA and are highly expressed in serum and synovium fluid of RA patients [22,23]. An increase in the concentration of T-cells and a reduction in CD4 native T-cells at inflammation sites determine the pathogenesis of RA [24]. When antigen-presenting cells interact with naive CD4 cells, T cells produce and proliferate into T regulatory cells and T helper cells. T regulatory cells and T helper cells release chemokines such as interleukin-17 (IL-17), interleukin-1β, interleukin-10 and interleukin-6. IL-17 stimulates the production of other immunological cells, such as macrophages, FLS, and cytokines (IL-1β, IL-6, and TNF-α) (see Figure 1) [25]. The production of these cytokines further stimulates the differentiation of T cells into (Th17) T helper cells. This differentiation is caused by the aryl hydrocarbon receptor (AhR), a transcription factor that is induced by IL-6 [26,27] Differentiation of naive T cells into T regulatory cells is also regulated by AhR. T regulatory cells have anti-inflammatory properties and produce anti-inflammatory cytokines such as TGF-β and interleukin-10 (IL-10) [28].

There are some drugs available that decrease inflammation and reduce pain, e.g., disease-modifying anti-rheumatic drugs (DMARDs), such as methotrexate, hydroxychloroquine, steroids, and non-steroidal anti-inflammatory drugs (NSAID); TNF inhibitors; and IL-6 inhibitors [8]. DMARDs are classified into conventional synthetic DMARDs (csDMARDs), targeted synthetic DMARDs (tsDMARDs), and biological DMARDs (bDMARDs). csDMARDs consist of leflunomide (LEF), sulfasalazine (SASP), and methotrexate (MTX); bDMARDs include inhibitors targeting B cells (rituximab), tumor necrosis factor (e.g., adalimumab and etanercept) T cells (abatacept), and interleukin-6 (IL-6; tocilizumab); and tsDMARDs consist of inhibitors of Janus kinase, baricitinib, and tofacitinib [29]. These medicines do have beneficial effects, but are also associated with a large number of side effects [11], which has prompted researchers to investigate new antiarthritic drugs. Herbal medicines have regained popularity, drawing researchers’ attention to them. Out of these, polyphenols are the most captivating and widely studied [12].

## 4. Biological Basis of Cancer Disease

Cancer is caused by the uncontrolled proliferation of normal cells in the body. Old cells do not demolish and continue to grow uncontrollably, resulting in the formation of new, abnormal cells. These uncontrolled cell divisions combine to form a mass of tissue known as a tumor. There are some physiological and biochemical factors that cause cancer, such as ionized and ultraviolet radiations, viral infections (e.g., human papillomavirus HPV causes cervical tumor growth and hepatitis B causes liver cancer), smoking, lack of exercise, high consumption of simple sugar and meat, parasites (e.g., schistosomiasis causes bladder cancer), contamination of meals or beverages (e.g., liver cancer may be caused by aflatoxins), and consumption of alcohol, which can cause liver cancer and bacterial infections (e.g., gastric cancer caused by *Helicobacter pylori*). Lung, stomach, colon, breast, prostate, and cervical cancers killed roughly 10 million individuals in 2020 [30,31,32,33,34]. For this reason, cancer therapies and preventive studies are necessary. There are many different phytochemical compounds present in fruits and vegetables that have anti-cancer, anti-inflammatory, antioxidant, and anti-diabetic properties. These are grouped into alkaloids, organosulfur compounds, carotenoids, vitamins, and phenolic compounds. Polyphenol-rich diets have anti-inflammatory, anti-cancer, anti-diabetic, cardioprotective, and anti-aging effects [22]. This review highlights the in vitro study of the chemopreventive effects of polyphenols against CD and RA.

## 5. In Vitro Test with Polyphenols

The Mediterranean diet has been known to reduce the incidence of chronic inflammation [32]. Extra virgin olive oil (EVOO) is one component of the Mediterranean diet which helps to reduce inflammation [33]. Phenolic compounds including tyrosol, hydroxytyrosol (HTyr), and oleuropein are key active components present in EVOO and have antioxidant and anti-inflammatory properties [34,35].

Many in vitro and in vivo studies have been performed to analyze the anti-arthritis effects of HTyr against various types of malignant cells, with different mechanisms of action being proposed. The investigation was conducted by Rosillo et al. to evaluate the efficacy of HTyr in a human synovial cell line, SW982. SW982 cells treated with HTyr had significantly reduced expression of tumor necrosis factors, matrix metalloproteinases, and IL-6. NF-jB and MAPKs phosphorylation activation induced by IL-1b was also inhibited by HTyr treatment (Table 1). These investigations suggest that HTyr can be a promising target for the prevention and management of RA [36].

The serine/threonine mitogen-activated protein kinases (MAPK) control the differentiation, growth, survival, and death of cells. MAPK signaling cascades consist of three kinases: the stress-activated protein kinase 2 (SAPK2), p38, c-Jun NH2-terminal kinase protein (JNK), and extracellular signal-regulated protein kinase (ERK1/2, p44/p42). P38 and JNK are activated by genotoxicity, hypoxia, oxidative stress, and cytokines. ERK is induced by cytokines and mitogens. MAPK signaling cascade is triggered in response to signals, which can be intra- or extracellular signals. In order to regulate target genes, these signals activate transmembrane glycoproteins of the tyrosine kinase receptor type.

**Figure 2:**
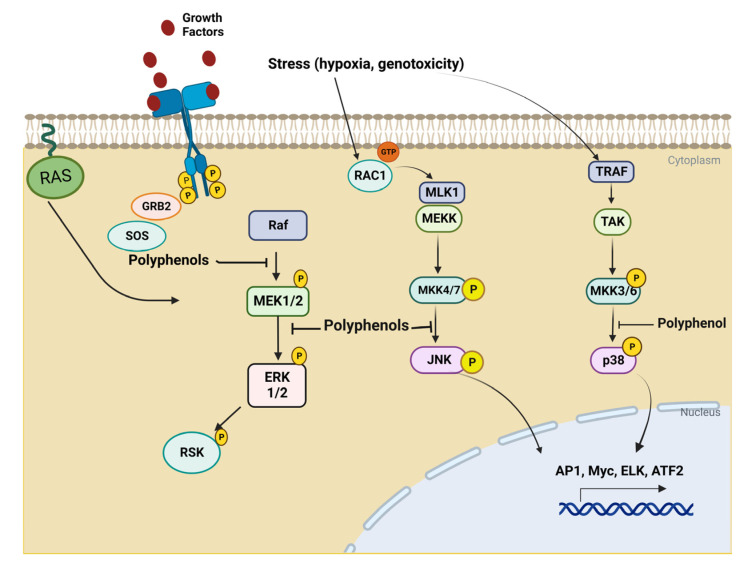
Potential sites of an inhibitory mechanism of polyphenols in MAPK signaling pathways. ERK, extracellular signal-related kinases; JNK, c-Jun amino-terminal kinases; p38, p38 mitogen-activated protein kinase.

**Figure 3:**
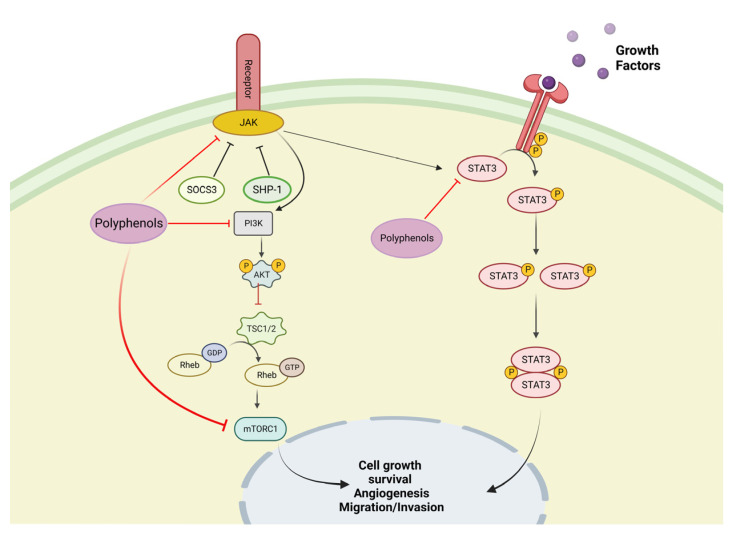
Potential sites of an inhibitory mechanism of polyphenols in the phosphatidylinositol 3-kinase (PI3K)/protein kinase B (Akt) and the mammalian target of rapamycin (mTOR) pathways.

The phosphatidylinositol 3-kinase (PI3K)/protein kinase B (Akt) and the mammalian target of rapamycin (mTOR) are signaling pathways involved in the regulation of cell growth and cell survival. These signaling pathways are activated by various stimuli and regulate various operations, such as transcription, translation, proliferation, growth and survival [37]. Polyphenols regulate the immune system by inhibiting the mitogen-activated protein kinase (MAPK), ERK, JNK, and p38 (Figure 2), as well as the PI3K/AKT, mTORC1, and JAK-STAT pathways (Figure 3). Polyphenolic compounds have been shown to affect the epithelial–mesenchymal transition (EMT) by upregulating epithelial markers, such as E-cadherin, and suppressing mesenchymal markers [38,39]. Natural polyphenols, including apigenin genistein, luteolin, resveratrol, and quercetin, have been proven to induce cell death in various cancerous cell lines [40]. In vitro studies have shown that polyphenol extracts modulate NF-κB and Nrf2 activation and regulate PI3K and MAPK function in cancer cell lines [41].

Olive oil (*Olea Europea* L.) is a well-known Mediterranean evergreen tree derivative with a slow growth rate and a life expectancy of up to 1000 years [42]. It is one of the most valuable trees for the Mediterranean economy, offering numerous commercial uses, such as in food, lumber, and cosmetics. The Mediterranean diet’s (MD) health benefits are known worldwide. Extra-virgin olive oil (EVOO) is increasingly considered a symbol of the MD. One of the significant differences between MD and other healthy diets is the high consumption of EVOO, which ranges from 15.3 to 23 kg per person per year [43]. EVOO-rich diet, with its omega-3 fatty acid content, is effective against many diseases such as Type 2 diabetes, RA, CD, and neurodegenerative and cardiovascular diseases [44,45].

The primary phenol classes in olive oil are phenolic acids, phenolic alcohols, flavonoids, secoiridoids, and lignans. Secoiridoids are a class of aglycon derivatives that contain oleanolic acid (EA) or derivatives of EA. The most abundant secoiridoids in olive oil are the dialdehyde forms of decarboxymethyl-EA linked to hydroxytyrosol (HT) or tyrosol (Tyr) (3,4-DHPEA-EDA and p-HPEA-EDA, respectively), oleuropein aglycone isomer (3,4-DHPEA-EA), and ligstroside aglycone (p-HPEA-EA) [46].

Phenolic acids are classified as hydroxybenzoic acid derivatives (p-hydroxybenzoic, protocatechuic, vanillic, syringic, and gallic acid) and hydroxycinnamic acid derivatives (p-coumaric, ferulic, cinnamic, caffeic, and synaptic acid) [47].

Hydroxy-isochrons are comprised of the only two molecules identified in commercial virgin olive oil, namely 1-phenyl-6,7-dihydroxy-isochron and 1-(3′-methoxy-4′ -hydroxy)-6,7-dihydroxy-isochron. These compounds are formed by the HTyr reaction with benzaldehyde and vanillin, respectively [48].

Flavonoids are made up of two benzene rings connected by three linear carbon chains. Further modifications, such as glycosylation, result in the formation of other compounds, which are classified as flavones, flavonols, flavanones, and flavanols. Flavones were the first flavonoids discovered in virgin olive oil; their free forms, luteolin, and apigenin are the most concentrated compounds [49]. Luteolin has anticancer properties under both in vitro and in vivo conditions. Luteolin hampers the processes of carcinogenesis, such as metastasis, cell transformation, and angiogenesis, through different pathways, e.g., inducing apoptotic cell death, regulating the cell cycle, reducing transcription factors, and suppressing kinases [50].

Lignans are distinguished chemically by the condensation of aromatic aldehydes. Lignans are found in the pulp of olives as well as the woody portion of the seed; these molecules are produced in the oil during the extraction process with no biochemical modifications. The lignans most abundant in EVOO are (+)-pinoresinol and (+)-1-Acetoxypinoresinol.

Phenolic alcohols (or phenylethanoids) have a hydroxyl group attached to an aromatic hydrocarbon group. The main molecules in this class are hydroxytyrosol (3,4-dihydroxy phenyl ethanol or 3,4 DHPEA; HTyr), tyrosol (p-Hydroxyphenyl ethanol or p-HPEA; Tyr), and oleocanthal. HTyr and Tyr are present in low concentrations in fresh olive oil, but their amounts increase significantly during storage due to the hydrolysis of secoiridoids [51].

Spices are used around the world in culinary culture and for the coloring, flavoring, and preserving of food, as well as for medicinal purposes. They have been reported to protect against many diseases, including neurodegenerative and cardiovascular diseases, type 2 diabetes, CD, and RA [52].

The grape (*Vitis vinifera*) is one of the world’s most significant and popular fruits. The consumption of grapes is linked to health benefits; it has anti-tumor and anti-diabetic properties. It is also effective in reducing the risk of cardiovascular disease [53]. Grapes have many polyphenolic compounds, including flavonols, anthocyanins, phenolic acid, and resveratrol. These polyphenolic compounds are present in the skin, leaves, stems, and seeds of grapes [54]. The total concentration of polyphenolic compounds present in seeds, skin, flesh and leaves are 2178.8, 374.6, 23.8, and 351.6 mg/g GAE (gallic acid equivalent). Proanthocyanidins are the major polyphenolic compounds contained in grapeseed and grape skin. Grapeseed contains the highest antioxidant compounds. [55]. However, the thick-skinned red grapes known as Cabernet Sauvignon have seed oil with the highest polyphenol content [56].

Various kinds of in vitro assays have been used for the evaluation of the anticancer and anti-arthritic activities of polyphenols. In this review, we described the in vitro assays used to examine cell apoptosis, viability, oxidative stress, cellular senescence, invasion, antioxidant effects, oxidative stress, angiogenesis, and gene and protein expression. Direct detection methods of factors affecting include real-time polymerase chain reaction (PCR), Western blotting, and enzyme-linked immunosorbent assay (ELISA) [57].

2′,7′-Dichlorofluorescein diacetate (DCFH-DA) is a probe used to quantify ROS and Nitric oxide (NO). Cells take DCFH-DA, and cellular esterase cleaves off the acetyl group, resulting in DCFH. DCFH is oxidized into DCF by ROSs, and it emits green fluorescence at the 485 nm wavelength and the emission wavelength of 530 nm [58]. Abbas et al. have also utilized this probe to quantify ROSs in Huh-7 human hepatocellular carcinoma cells treated with curcumin [59].

DNA oxidative damage ELISA kits are used to assess 8-hydroxy-2′-deoxyguanosine (8-OH-dG) levels as an oxidative stress marker [60]. Immunofluorescent staining antibodies were carried out to detect the effects of pterostilbene on the reduction in oxidative damage induced by hyperosmotic stress in human corneal epithelial cells in a study conducted by Colin et al. 3-(4,5-dimethylthiazol-2-yl)-2,5-diphenyltetrazolium bromide (MTT) cell proliferation assays have commonly been employed to assess the proliferation of cells [61]. An endothelial cell (HUVEC) proliferation assay was used in the study conducted in [40]. Chang et al. have also conducted cell proliferation assays with human synovial cell lines [62]. The fluorescent microscopic technique has been used to detect morphological changes upon treatment with various polyphenols in various cancer and RA cell lines [63,64]. It has become an excellent technique for cell and tissue imaging and to observe biological processes.

The tube formation assay was first explained by Kubota et al. in 1988. It is used for in vitro assays for angiogenesis. The procedure of this assay involves the plating of endothelial cells on the basement of a membrane-like substrate. Cells create cell-to-cell and cell-to-matrix attachments. The tube area or length of the branch point can be quantified using this assay [65].

### 5.1. In Vitro Test with Olive Oil Polyphenols

#### 5.1.1. In Vitro Test with Tyrosol on Rheumatoid Arthritis Cellular Models

Tyrosol is phenethyl alcohol, and is present in olive oil. It has anti-inflammatory and antioxidant properties. Luo et al. found that tyrosol reduces the release of IL-6 and TNF-α in cerebral hippocampal astrocytes isolated from post-neonatal pups of C57BL/6j mice. The decreased expression of cytokines is due to astrocyte inhibition and STAT3 signaling pathway regulation. Tyrosol also inhibits the IκBα degradation and enhances the phosphorylation of IκBα, leading to the downregulation of NF-κB expression [66]. Kim et al. concluded that tyrosol decreased the expression of inducible nitric oxide synthase (iNOS), cyclooxygenase (COX)-2, and phosphorylated-IκBα in LPS-stimulated RAW 264.7 macrophages [67]. These outcomes show that tyrosol modulates the inflammatory response and can be used as a treatment compound for RA.

#### 5.1.2. In Vitro Test with Oleocanthal on Rheumatoid Arthritis Cellular Models

Oleocanthal is a polyphenolic compound, an important constituent of extra virgin olive oil, with anti-inflammatory properties. Studies by Scotece et al. (2012) examined that oleocanthal inhibits lipopolysaccharide-induced nitric oxide production and expression of iNOS, and suppresses the expression of macrophage inflammatory protein-1α. Oleocanthal also inhibits the production of IL-6 in J774 macrophages and ATDC5 chondrocytes, as well as the production of IL-1β, TNF-α, GM-CSF, and NO in J774. These cytokines play a significant role in the inflammatory process and destruction of cartilage in RA [68]. This research indicates that oleocanthal has anti-inflammatory properties and can be used for the treatment of RA.

#### 5.1.3. In Vitro Test with Oleuropein on Rheumatoid Arthritis Cellular Models

Oleuropein is the most important phenolic compound in olive oil, with antioxidant and anti-inflammatory properties. It is used as a food supplement in Mediterranean countries. Its anti-inflammatory characteristics were evaluated by Castejón et al. The effects of oleuropein were evaluated on the IL-1β-induced human synovial sarcoma cell line (SW982). It was observed that the expression of inflammatory cytokines IL-6, TNF-α, MMP-1, MMP-3, mPGES-1, and COX-2 were decreasing. This outcome proves that oleuropein can be used for the management and prevention of RA [69].

#### 5.1.4. In Vitro Test with Hydroxytyrosol on Cancer Cell Lines

The authors in [70] showed that hydroxytyrosol induces apoptosis in the LS180 colorectal cancer cell line by upregulation of pro-apoptotic genes such as BAX, CASP3, and P53, and also increases the BAX:BCL2 ratio and decreases nuclear factor erythroid 2-related factor 2 (NFE2L2) expression. Furthermore, hydroxytyrosol treatment increases antioxidative activity in colorectal cancer-cell lines, as evidenced by increased antioxidant enzymes. In another study [50], the authors demonstrated that HTyr can induce apoptosis in DLD1 colon cancer cells by producing ROSs. ROSs activated the PI3K/AKT/FOXO3 pathway, which regulated FOXO3 targets such as SOD and catalase, contributing to a reduction in cellular antioxidant defenses. In vitro studies of HTyr’s effects on colon cancer proliferation proposed that olive oil phenolic extracts regulate epigenetic mechanisms. CpG island methylation on the promoter of the Type I Cannabinoid Receptor (CB1), which could function as a tumor suppressor, has been frequently reported in the context of various cancers, including colon cancer. In vitro, the administration of olive oil phenolic extracts, including oleuropein and HTyr, regulates CB1 gene expression by lowering the methylation status of its promoter and lowering tumor cell proliferation [71]. A recent study suggests that HTyr reduces hypoxia-inducible factor-1 (HIF-1) in MCF-7 breast cancer cell lines by lowering oxidative stress and inhibiting the P13K/Akt/mTOR pathway. HTyr also upregulates the expression of vascular endothelial growth factor, even in HIF-1 silenced cells [72].

#### 5.1.5. In Vitro Test with Luteolin on Cancer Cell Lines

An investigation conducted in 2015 by Sun et al. demonstrated that the luteolin inhibits MDA-MB-231 breast cancer cell survival, as well as the expression of Notch signaling-related protein and mRNAs [73]. The 2015 study conducted by Jeon et al. showed that luteolin expresses chemopreventive properties in MCF-7, HER18, MDA-MB-231, and SkBr3 cells by inhibition of extracellular signal-regulated kinase (ERK) via Akt inactivation [74]. In another study, the authors revealed that luteolin inhibits the activation of MAPK signaling pathway 9 in 12-o-tetradecanoylphorbol-13-acetate (TPA)-treated breast cancer cells (MCF-7), leading to downregulation of the expressions of IL-8 and MMP. Luteolin also downregulates the activation of the AP-1 and NF-κB pathways [75].

M. J. Kim et al. described that the luteolin activates p38 and ERK. Luteolin induces nuclear translocation of apoptosis-inducing factors (AIFs), mediated by the activation of p38 and ERK [76]. In another study, the authors revealed that luteolin downregulated the expression level of VEGF by inhibiting NF-κB in human pancreatic carcinoma cell lines (PANC-1) [77]. Research by Pratheeshumar et al. has shown that luteolin inhibits VEGF-A-induced phosphorylation of VEGF receptor 2, as well as protein kinases ERK, A, KT, and mTOR [78].

### 5.2. In Vitro Test with Spice Polyphenols

#### 5.2.1. In Vitro Test with Curcumin Polyphenols on Rheumatoid Arthritis Cellular Models

Curcumin is a bright yellow chemical produced by the *Curcuma longa* species which is a natural anti-inflammatory agent. Curcumin has been demonstrated to have anti-inflammatory activities by blocking the COX-2 pathway. It has also been observed that curcumin decreases the production of vascular endothelial growth factor (VEGF) and IL-6. It also inhibits the extracellular signal-regulated kinase (ERK1/2) and NF-κB inflammatory pathways [79].

The production of receptor activators of nuclear factor κB ligand (RANKL) and osteoclast-associated RANK is important for the process of osteoclastogenesis. RANK binds to RANKL, and as a result, osteoclast differentiation begins. Inflammatory cytokines upregulate the expression levels of RANK, enhance osteoclast precursors, and increase their sensitivity to RANKL, which may result in bone erosion in RA. Curcumin may inhibit the osteoclastogenic potential of PBMCs in patients with RA through the suppression of the mitogen-activated protein kinase/RANK/c-Fos/NFATc1 signaling pathways. NFATc1 is a crucial transcription factor that is expressed in osteoclast precursors through Ca^2+^ oscillation, MAPKs, and c-Fos or RANK in response to RANKL [80]. In the study [81], the authors highlighted that high expression of IL-6 is due to histone modification in RA synovial fibroblasts. Histone H3 acetylation level was higher in the IL-6 promoter region. Curcumin inhibited histone acetyltransferase, resulting in a reduced expression level of H3ac in the IL-6 promoter region, and ultimately expressed a reduced level of IL-6. Curcumin decreased cell proliferation and interferon-gamma (IFN-γ) production in TEM cells in patients with RA [82]. One study conducted in 2014 showed that curcumin enhances the expression of miR-181b and decreases the expression level of CXCL1 and CXCL2 (pro-inflammatory chemokines) [83].

#### 5.2.2. In Vitro Test with Curcumin Polyphenols on Cancer Cell Lines

Curcumin is an essential natural chemical that has anti-inflammatory and anti-tumor activities. The chemopreventive effects of curcumin were investigated in cultured breast cancer cells. Specifically, curcumin was found to suppress the proliferation of several breast cancer cell lines, including T47D, MCF7, MDA-MB-231, and MDA-MB-468 [84]. Curcumin also suppressed protein kinase B (Akt)/mammalian target of rapamycin (mTOR) phosphorylation, decreased B-cell lymphoma 2 (BCL2), and enhanced BCL-2-associated X protein (BAX) and caspase 3 cleavage, resulting in apoptosis in breast cancer cells [85]. Curcumin suppressed breast cancer cell growth and triggered G2/M phase cell cycle arrest and apoptosis, which could be linked to decreased CDC25 and CDC2 protein levels, increased P21 protein levels, suppression of Akt/mTOR phosphorylation, and stimulation of the mitochondrial apoptotic pathway [86].

Studies by Liu et al. examined curcumin’s powerful inhibitory effects on breast cancer, the most common malignancy in women around the world. Breast cancer cell lines that express estrogen receptors (ER) have a low maximal inhibitory concentration, which sensitizes them to anti-cancer medicines. Furthermore, these treatments can cause apoptosis in cell lines regardless of hormone receptor expression. Curcumin also inhibits the multiplication of breast cancer stem cells (BCSC), which has a key role in cancer recurrence. BCSC proliferation suppression inhibits metastasis and reattachment, thus restricting tumor development. Curcumin suppresses tumor growth in cancer cells and cancer stem cells, according to a xenograft study. As a result, curcumin looks to be a promising anticancer chemical when used in combination with other anticancer treatments [84].

Curcumin inhibits endometrial carcinoma (EC) cell invasion and migration. It was observed that the curcumin decreases the expression of matrix metalloproteinase-2 as well as matrix metalloproteinase-9. It was also observed that curcumin reduces the expression level of extracellular signal-regulated kinase (ERK) [87].

#### 5.2.3. In Vitro Test with Ginger Polyphenols on Cancer Cell Lines

Ginger (*Zingiber officinale*) is a domesticated spice that is used as a food additive. It is used in herbal medicine and has many medicinal properties. Ginger has many bioactive components, including anthocyanins, volatile oils, tannins, sesquiterpenes, and gingerols [88]. Gingerol is an active ingredient in ginger with anti-cancer properties, and it modulates various signal pathways in cancerous cells, i.e., nuclear factors (NF-KB), signal transducer and activator of transcription 3 (STAT3), activator protein-1 (AP-1), wnt/β-catenin, growth factor receptors (EGFR, VEGFR), mitogen-activated protein kinases (MAPK), and pro-inflammatory mediators [89]. In one study, the anti-cancer activity of 6-Gingerol was evaluated on the 143B Human osteosarcoma cell line. The viability of osteosarcoma cells was decreased after treatment with 6-Gingerol, which activates the AMP-activated protein kinase pathways and caspase cascades and regulates the levels of Bcl2 and Bax, resulting in the apoptosis of cells [90]. Similarly, in another study, human colon adenocarcinoma (SW-480) cells were treated with [6]-gingerol activate caspase-3 and -7, with cleavage of procaspase-3 and -7a, and, therefore, caspase-mediated apoptosis was confirmed [91].

The effect of 6-Gingerol on adenocarcinoma human alveolar basal epithelial cells (A549 cells) showed that 6-Gingerol inhibits cell proliferation and survival through the autophagy-ferroptosis pathway treatment, resulting in a decrease in tumor volume, but also in a decrease in accumulation of ROS and iron in the tumor. Lower expression levels of autophagy- and ferroptosis-related proteins are caused by suppressing the expression of Ubiquitin-specific protease 14 (USP14) [92], so it is suggested that 6-Gingerol could be a potential natural drug against cancer.

#### 5.2.4. In Vitro Test with Stilbenes on Rheumatoid Arthritis Cellular Models

Stilbenes are polyphenolic non-flavonoid compounds found in berries, grapes, red wine, peanuts, etc., which have anti-inflammatory and antioxidant properties. More than 400 stilbene compounds have been identified. Resveratrol is an important compound of stilbenes that is present in the outer layer of the skin of grapes [93] and that increases the expression of heme oxygenase-1 (HO-1) and nuclear factor erythroid 2-related factor 2 in H_2_O_2_ treated (RA-FLS) RA fibroblast-like synoviocyte cells. Furthermore, it also downregulates the expression of kelch-like ECH-related protein 1 (keap1), ROS, and MDA. It also blocks the expression of nuclear factor-κB (NF-κB) p65 and enhances the expression of Bcl-2/Bax, which leads to inhibition of cell proliferation and apoptosis [94]. It has been determined that resveratrol induces cell apoptosis in the MH7A cell line by activation of caspase-9 and caspase-3 and disruption of mitochondria. Disruption of mitochondria causes the downregulation of the expression of Bcl-XL and the release of cytochrome c into the cytosol from mitochondria [95]. It has also been proven that resveratrol modulates the production of cytokines and inhibits the protein expression of MMP-3 and IL-1b in fibroblast-like synoviocytes. Resveratrol also inhibits the TNF-a-stimulated production of P-Akt. These findings suggest that resveratrol has anti-inflammatory properties and can be used for the prevention and treatment of RA [11].

Kaempferitrin is a flavonoid glycoside which has anti-inflammatory properties. One study showed that it was able to reduce the levels of IL-6, IL-1 β, MMP-1, MMP-3, and TNF-α in MH7A cell lines. It inhibits the activation of protein kinase B and nuclear factor-κB (NF-κB) [57]. Another study analyzed kaemperitin, which inhibits the activation of NF-jB and MAPK in IL-1β-stimulated RASFs, as well as the expression levels of COX-2, MMP-1, MMP-3, and PGE2 [96].

Yoon et al. treated RA-FLS with gallic acid. The results showed that gallic acid inhibits the expression of different chemokines, proinflammatory cytokines, MMP-9, and COX-2 and induces cell apoptosis by regulation of Bcl-2, caspase-3, p53, and Bax [97].

Sung et al. conducted a study and concluded that the quercetin suppresses the proliferation of IL-1β-stimulated and unstimulated rheumatoid synovial fibroblasts (RASFs). It inhibits the expression of PGE2, COX-2, and MMPs by inhibiting various signaling pathways, including ERK1/2, p-38, JNK, NF-kB, and MAP kinases. These results show that quercetin can be utilized for the prevention and management of RA [98].

Kitamura et al. explained that Heme oxygenase 1 (HO-1) is highly expressed in the synovial fluid of RA patients [99]. HO-1 is also highly regulated in rat adjuvant-induced arthritis models and murine collagen-induced arthritis [100,101]. Heme oxygenase is a microsomal enzyme with anti-inflammatory and antioxidant functions. It degrades the heme group and yields free iron, biliverdin, and carbon monoxide [102]. One study, investigated whether quercetin had antioxidant properties. It was found that it activated the MAPK–Keap1–Nrf2–ARE signaling pathways, which are responsible for the expression of the HO-1 gene [103].

Durromond et al. treated THP1 macrophages with apigenin, quercetin, and salicylic acid. Both apigenin and quercetin decreased the expression of IL-6 and TNF-α [104].

### 5.3. In Vitro Test with Grapes Polyphenols

#### In Vitro Test with Resveratrol on Cancer Cell Lines

Studies by Jang et al. examined the anti-tumor activity of resveratrol on androgen-sensitive human prostate adenocarcinoma cells (LNCaP), a human prostate cancer cell line. Prostate cancer is the second-most common cause of cancer-related mortality. Studies have shown that prostate cancer is affected by the action of dihydrotestosterone on androgen receptors. C-X-C chemokine receptor type 4 (CXCR4) is a receptor that is highly expressed in prostate cancer cells. Dihydrotestosterone proliferates LNCaP prostate cancer cells. The results showed that resveratrol and its combination with AMD3100 (CXCR4 inhibitor) reduced the cell viability promoted by dihydrotestosterone [105]. Studies conducted by Aires et al. concluded that 3-o-sulfate-Resveratrol, a metabolite of resveratrol, inhibits human colon cancer cell lines due to S-phase stem cell accumulation, the apoptosis process, and DNA damage to the colon [106]. Feng et al. observed that resveratrol downregulated the expression of cyclooxygenase 2 (COX-2) in the HT29, SW480, and HCA-17 cell lines [107]. Cyclooxygenases convert arachidonic acid into prostaglandins. Many carcinoma cells, such as breast, pancreatic, colon, hepatic, and gastric cancer, have high expression levels of COX-2. Arachidonic acid induces cell death without the involvement of prostaglandins. Conversion of arachidonic acid into prostaglandins via COX-2 high expression inhibits cell apoptosis. Cyclooxygenase-1 and -2 promote angiogenesis, while prostaglandin-2 inhibits cell apoptosis via Bcl-2 expression. The study conducted by Feng et al. demonstrated that the resveratrol-treated human colon cancer cell lines showed low prostaglandin and clyclooxygenase-2 receptor expression.

Ko et al. observed that resveratrol inhibits the expression of XRCC1 in non-small-cell lung cancer (NSCLC). XRCC1 is a scaffold protein that plays an important role in base excise repair by regulation of AKT and ERK1/2 signals and, therefore, has a role in the progression of lung cancer [108].

Resveratrol promotes cell apoptosis by inducing caspase-8- and -3-dependent apoptosis via ROS-triggered autophagy in COLO 201 and HT-29 human colon cancer cells [109].

According to the observations of Colin et al., resveratrol (30 µM) overproduces ROS in colon cancer cells [110]. Demoulin et al. suggested that resveratrol induces DNA damage due to overexpression of topoisomerase II [111].

Resveratrol inhibits metastasis in hepatocellular carcinoma cells. It also downregulates the expression of urokinase-type plasminogen activators, which inhibits the SP-1 signaling pathway and is also a powerful chemopreventive agent against liver cancer. At low concentrations, resveratrol treatment (25–100 µM) inhibited the metastasis of HCC cells and decreased expression of urokinase-type plasminogen activator (u-PA), which involved downregulation of the SP-1 signaling pathway [112]. Gracia-Zepeda et al. explained that resveratrol arrests the cell cycle at the G1 phase, and induces apoptosis and lysomosal permeability in HeLa, SiH, Caski, and C33A cell lines [113]. These studies prove that resveratrol can be used for the treatment of various kind of cancers. Table 1 presents the summary of documents published on polyphenols used in vitro which have been discussed in this review.

**Table 1:** Summary of state-of-the-art polyphenols used in vitro which have been discussed in this review.

Polyphenols	Cell Lines	Major Findings	References
Rheumatoid Arthitis			
Hydroxytyrosol	SW982	Decrease the expression of TNF, MMP and IL-6	[114]
Tyrosol	RAW 264.7 macrophages	Decrease the release of inducible iNOS, COX-2, and phosphorylated-IκBα	[67]
Oleocanthal	J774 macrophages and ATDC5 chondrocytes	Inhibition of expression of iNOS and IL-6, and suppression of the expression of macrophage inflammatory protein-1	[68]
Oleuropein	SW982	Inhibition of cytokines IL-6, TNF-α, MMP-1, MMP-3, mPGES-1, and COX-2 expression	[69]
Curcumin	MH7A, FLS	Decrease in the production of VEGF and IL-6; inhibition of the ERK1/2 and NF-κB inflammatory pathways	[79]
Resveratrol	RA-FLS, MH-7A	Inhibition of the TNF—α—stimulated production of P-Akt, activation of NF-jB expression, increase in the expression of HO-1and NRF2 Downregulation of the expression of kelch-like ECH-related protein 1 (keap1), ROS, and MDA. Arrest of the expression NF-κB p65 and enhancement of Bcl-2/Bax expression. Activation of caspase-9 and caspase-3. Inhibition of the of MMP-3 and IL-1b protein expression.	[11,104,105]
Kaempferitrin	MH7A, RASFs	Decrease of IL-6, IL-1 β, MMP-1, MMP-3, COX-2, PGE2, and TNF-α levels. Inhibition of the activation of protein kinase B and NF-κB., NF-jB, and MAPK.	[65,106]
Gallicacid	RA-FLS	Inhibition of the expression of different chemokines, proinflammatory cytokines, MMP-9, and COX-2, as well as induction of cell apoptosis by regulation of Bcl-2, caspase-3, p53, and Bax.	[97]
Quercetin	RASFs	Inhibition of the expression of PGE2, COX-2, and HO-1 genes, as well as MMPs, by inhibiting various signaling pathways, including ERK1/2, p-38, JNK, NF-kB, and MAPK.	[108,113]
Cancer Disease			
Hydroxytyrosol	LS180 MCF-7	Upregulating pro-apoptotic genes such as BAX, CASP3, and P53, as well as increasing the BAX: BCL2 ratio and decreasing NFE2L2 expression, lowering oxidative stress, and inhibiting the P13K/Akt/mTOR pathway.	[80,82]
Luteolin	MCF-7	Inhibition of MDA-MB-231, activation of the MAPK signaling pathway, expression of Notch signaling-related protein and mRNAs. Inhibition of ERK via Akt inactivation and others; inhibition of 9 in TPA.	[62,63,64]
Curcumin	T47D, MCF7, MDA-MB-231, and MDA-MB-468	Suppresses of protein kinase B (Akt)/mTOR phosphorylation, decreased BCL2, and enhancement of BCL-2-associated X protein (BAX) and caspase 3 cleavage. Triggers G2/M phase cell cycle arrest and apoptosis, increasex P21 protein levels, suppresses Akt/mTOR phosphorylation, and stimulates the mitochondrial apoptotic pathway.	[73,96]
Gingerol	Human osteosarcoma cell line 143B.	Modulation of various signal pathways in cancerous cells, e.g., NF-KB, STAT3AP-1EGFR, VEGFRMAPK, and pro-inflammatory mediators. Activation of the AMP-activated protein kinase pathways and caspase cascades, and regulation of Bcl2 and Bax levels, resulting in the apoptosis of cells.	[77,100]
Resveratrol	LNCaP, HT29, SW480, and HCA-17 cell lines	Promotion of the accumulation of cells in S-phase, apoptosis process, and DNA damage. Inhibition of the receptor expression of XRCC1.	[106,107]

** nitric oxide synthase (iNOS), cyclooxygenase (COX)-2, X-ray repair cross-complementing protein-1 (XRCC1), nuclear factors (NF-KB), extracellular signal-regulated kinase (ERK), signal transducer and activator of transcription 3 (STAT3), activator protein-1 (AP-1), -catenin, growth factor receptors (EGFR, VEGFR), mitogen-activated protein kinases (MAPK), B-cell lymphoma 2 (BCL2), 12-o-tetradecanoylphorbol-13-acetae (TPA), nuclear factor erythroid-derived 2-like (NEF2L2), vascular endothelial growth factor (VEGF), extracellular signal-regulated kinase (ERK1/2), heme oxygenase-1 (HO-1), mammalian target of rapamycin (mTOR).

## 6. Conclusions

**Figure 4:**
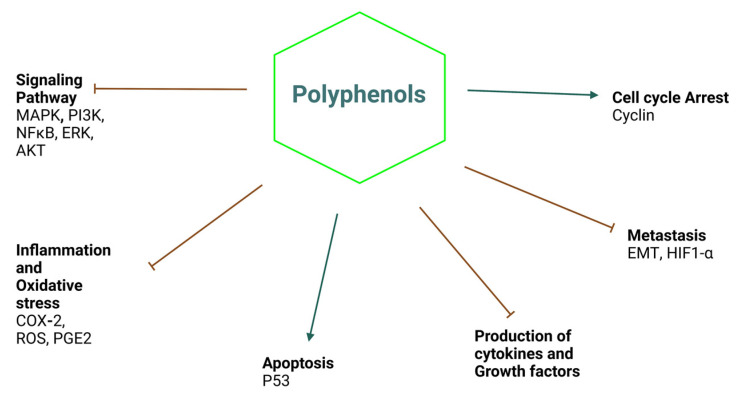
The figure schematically elucidates the overall summary of the review.

Herbal medicine is an ancient remedy, but it is gaining researchers’ attention due to its potential biological activities. These phytochemicals have the ability to prevent and treat certain diseases. This review highlights the chemopreventive and anti-arthritic properties of natural polyphenols, which have been proven to be effective in preclinical and clinical trials. The most studied polyphenols are hydroxytyrosol, quercetin, and chalcones. They can modulate various signaling pathways related to proliferation, differentiation, cell survival, apoptosis, angiogenesis, and immune responses. They are also able to inhibit inflammatory pathways in fibroblast cell lines; activate signal transduction pathways such as transcription factors and kinases, oncogene expression, and cell proliferation; induce tumor suppressor gene expression and cell cycle phases; and inhibit various signaling pathways, including NF-jB, AP-1, Nrf-KEAP1, and MAPK (Figure 4). They also suppress the production of autoantigens and antibodies responsible for the development of RA. Studies in both in vitro and in vivo conditions are needed to understand the physiological, biodistribution, and biological mechanisms of these compounds. The need to develop well-designed, targeted phenolic compounds for the treatment and prevention of diseases, or to combine these compounds with other drugs to enhance their therapeutic effects, is great.
